# Hypothermia protects brain mitochondrial function from hypoxemia in a murine model of sepsis

**DOI:** 10.1177/0271678X15606457

**Published:** 2015-11-26

**Authors:** Kim I Chisholm, Keila K Ida, Andrew L Davies, Ilias Tachtsidis, Dmitri B Papkovsky, Alex Dyson, Mervyn Singer, Michael R Duchen, Kenneth J Smith

**Affiliations:** 1 Institute of Neurology, University College London, UK; 2 Anaesthesiology LIM-8, Medical School, University of São Paulo, Brazil; 3 Medical Physics and Biomedical Engineering, University College London, UK; 4 School of Biochemistry and Cell Biology, University College Cork, Ireland; 5 Bloomsbury Institute of Intensive Care Medicine, University College London, UK; 6 Cell and Developmental Biology, University College London, UK

**Keywords:** Imaging, hypothermia, microcirculation, inflammation, microscopy

## Abstract

Sepsis is commonly associated with brain dysfunction, but the underlying mechanisms remain unclear, although mitochondrial dysfunction and microvascular abnormalities have been implicated. We therefore assessed whether cerebral mitochondrial dysfunction during systemic endotoxemia in mice increased mitochondrial sensitivity to a further bioenergetic insult (hyoxemia), and whether hypothermia could improve outcome. Mice (C57bl/6) were injected intraperitoneally with lipopolysaccharide (LPS) (5 mg/kg; *n* = 85) or saline (0.01 ml/g; *n* = 47). Six, 24 and 48 h later, we used confocal imaging *in vivo* to assess cerebral mitochondrial redox potential and cortical oxygenation in response to changes in inspired oxygen. The fraction of inspired oxygen (FiO_2_) at which the cortical redox potential changed was compared between groups. In a subset of animals, spontaneous hypothermia was maintained or controlled hypothermia induced during imaging. Decreasing FiO_2_ resulted in a more reduced cerebral redox state around veins, but preserved oxidation around arteries. This pattern appeared at a higher FiO_2_ in LPS-injected animals, suggesting an increased sensitivity of cortical mitochondria to hypoxemia. This increased sensitivity was accompanied by a decrease in cortical oxygenation, but was attenuated by hypothermia. These results suggest that systemic endotoxemia influences cortical oxygenation and mitochondrial function, and that therapeutic hypothermia can be protective.

## Introduction

Sepsis is estimated to be the 11th most common cause of death^1^ with a rising incidence due to an increasingly aged and immunosuppressed population.^2^ Septic patients commonly experience brain dysfunction, ranging from mild confusion to coma, and this is associated with increased mortality.^3^ Despite its prevalence and severity, the mechanisms by which sepsis leads to encephalopathy are still unclear. Sepsis is associated with mitochondrial dysfunction in various organs including skeletal muscle,^4–6^ liver,^4,5,7–9^ and kidney.^10^ Increasing evidence suggests that bioenergetic failure plays an important role in the pathogenesis of organ failure though this has not been definitively proven. Indeed, it has been suggested that patients may die not from the initial infection *per se* but rather because of factors that persist even after the infection has been successfully treated, such as a pronounced energy imbalance^11^. Additionally, sepsis has been associated with microvascular disturbances and poor organ perfusion^12–22^ with reports of reduced cerebral blood flow in patients^23,24^ and animal models.^25,26^ However, other studies have failed to detect any abnormalities.^27,28^

If mitochondrial dysfunction and bioenergetic failure do play a key role, states which compromise energy supply further (such as hypoxemia) would be expected to be particularly detrimental in sepsis, whereas conditions that reduce energy demand (such as hypothermia) could provide protection. Indeed, hypothermia has been successfully used in the treatment of several critical care conditions and has shown potential neuroprotective benefits.^29,30^ However, a recent clinical study of therapeutic hypothermia in bacterial meningitis was prematurely abandoned due to increased risk of mortality.^31^ In murine models of sepsis, hypothermia has a rapid onset and the magnitude of change in body temperature is related to reduced oxygen consumption and increased mortality rates.^32^ Thus, the potential role of therapeutic hypothermia remains uncertain.

We and others have previously shown that the endogenous green fluorescence of flavoproteins can be used to assess mitochondrial redox potential *in vivo* in the central nervous system^33,34^ (for review, see Reinert et al.^35^). Here, we have used this technique to assess changes in cortical mitochondrial sensitivity to an energetic challenge (hypoxemia) in a murine model of endotoxic sepsis, induced via injection of the lipopolysaccharide (LPS) component of the Gram-negative bacterial cell wall to provoke a potent inflammatory response. As such, LPS-induced endotoxemia can be used to model the inflammatory component of sepsis. We therefore used this model to assess cortical mitochondrial vulnerability during systemic inflammation, and the efficacy (or otherwise) of hypothermia in protecting mitochondrial function within the cerebral cortex of endotoxic mice during hypoxemia.

## Material and methods

Inbred mice (C57bl/6 strain, approximate 20 g body weight) were housed on a 12 h light/dark cycle with food and water available *ad libitum*. All experiments were approved by the ethics committee of the University College London, Institute of Neurology, and licenced by the UK Home Office under the Animals (Scientific Procedures) Act of 1986. The ARRIVE guidelines were followed where appropriate.

Mice were injected with LPS (from *Escherichia coli* 0111:B4; Sigma-Aldrich, Poole, UK; i.p. 5 mg/kg in sterile normal saline, 0.01 ml/g) or normal saline alone, and then returned to their cages until terminal surgery. The mice were randomized into nine groups for imaging and four groups for oxygen measurements (Table 1). The experimenter was not blinded. As the results showed a recovery from LPS-induced endotoxemia at 48 h, hypothermia treatment was not tested at this time point. Additionally, induced hypothermia (32℃) was only applied to endotoxic and saline control mice imaged 6 hours after injection, as the induced hypothermia condition was studied to understand the effect of hypothermia on healthy mice.

**Table 1.** Experimental conditions and numbers of animals used for flavoprotein imaging and oxygen probe experiments.

		6 h post-LPS	24 h post-LPS	48 h post-LPS	Saline
Flavoprotein imaging	Normothermia (37℃)	17	13	7	21
	Spontaneous temperature	13	13	-	10
	Induced hypothermia (32℃)	13	-	-	10
Oxygen probe measurements	Normothermia (37℃)	3	3	3	6

Mice were terminally anesthetized using isoflurane (∼2% in room air) either 6, 24 or 48 h after LPS injection as indicated (see Table 1). From this point onwards, rectal temperature was measured and controlled via a rectal probe and a homeothermic heating mat maintained at either 37℃ (*normothermia*), 32℃ (*induced hypothermia*), or the animal’s spontaneous rectal temperature (*spontaneous hypothermia*). An incision was made in the scalp and connective tissue removed. A titanium bar was affixed to the skull using dental cement (Contemporary Ortho-Jet Powder, Lang Dental, Wheeling, US) mixed with cyanoacrylate glue (Loctite, Henkel Ltd., Hatfield, UK). After the dental cement had dried (∼10 min), a partial craniotomy was performed over the right hemisphere (∼5 mm in diameter) exposing the underlying cortex. The dura was kept intact and moistened and cleaned with saline. In a subset of mice (*n* = 27), oxygen-sensitive microbeads impregnated with a phosphorescent dye, platinum(II)-5,10,15,20-tetrakis(2,3,4,5,6-pentafluorophenyl)porphyrin (PtPFPP; kindly provided by Luxcel Biosciences, Cork, Ireland) were spread on the dura (5 µl of 5 mg/ml aqueous suspension). A 6 mm diameter circular coverslip was placed over the exposed cortex and sealed with petroleum jelly to prevent evaporation of liquid during imaging.

After surgery, mice were moved to a custom-made stage on a LSM 5 Pascal laser-scanning confocal microscope (Carl Zeiss, Jena, Germany). Endogenous flavoprotein fluorescence (excitation: 488 nm, emission: 505–570 nm) and oxygen-sensitive microbeads (excitation: 543 nm, emission: 650 nm, collected with 585 nm long pass filter) were imaged using time series recording with an in-plane resolution of 512 by 512 pixels and an optical slice thickness of 896 µm. During imaging, FiO_2_ was maintained for 5-min intervals in sequence as follows: 0.21, 1.0, 0.21, 0.15, 0.21, 0.10, 0.21, 0.05 until death. Room air was delivered via an air pump while variations in FiO_2_ were administered as a mixture of oxygen and nitrogen.

For cortical oxygen tension studies, mice were anesthetized as above using isoflurane (∼2% in room air). Rectal temperature was measured and controlled via a rectal probe and a homeothermic heating mat at 37℃. A partial craniotomy was performed over the right hemisphere (approximately 2 mm). The dura mater was removed and the fiber-optic tip of an oxygen microsensor (OxyMicro, World Precision Instruments, Hitchin, UK) was inserted into the cortex using a micromanipulator in order to detect luminescence quenching. The probe was inserted to a depth of 700 µm followed by a retraction of 100 µm to a final recording depth of 600 µm from the cortical surface. Cortical tissue oxygen tension (PtO_2_) was measured continuously during changes in inspired oxygen as follows: 0.21, 0.15, 0.21, 0.10, 0.21, 0.05, for 5 min each, until death.

Heart rate was recorded in a subset of animals (*n* = 53) using conventional electrophysiological equipment (Neurolog, Digitimer, Welwyn Garden City, Herts, UK). A reference electrode (hypodermic needle) was inserted in the right hind foot, a ground electrode was inserted subcutaneously into the back, and a recording electrode was inserted subcutaneously in the chest wall. The ECG record was displayed on a digital oscilloscope (Sigma 60, Nicolet Technologies, Madison, WI, USA) and heart rate recorded.

Confocal images were processed using Fiji/ImageJ Version 1.48v (NIH, Bethesda, MD). Following alignment of time lapse recordings (using the ‘Stackreg’-Plugin), three representative areas immediately adjacent to arteries (periarterial) and three areas adjacent to veins (perivenous) were free-hand selected in each time-lapse series for each animal (see Figure 2(a) for example selections) and the fluorescence intensity normalized to the initial room air condition for each area. The ratio of periarterial and perivenous fluorescence intensity during the last five cycles of each FiO_2_ manipulation (0.21, 1.0, 0.21, 0.15, 0.21, 0.10, 0.21, 0.05) were recorded until death. Up to eight oxygen-sensitive phosphorescent beads were selected per time-lapse and their fluorescence intensity normalized to the initial room air conditions. The normalised intensity was then determined for the last five cycles of the FiO_2_ manipulations, as with flavoprotein fluorescence. Cortical tissue oxygenation, as recorded by the oxygen sensor, was averaged over the last minute of the FiO_2_ manipulations.

Data are displayed as mean ± standard error of the mean (SEM). Statistical analysis between experimental groups was conducted using the IBM SPSS Statistics 22 package (SPSS, Chicago, IL, USA).

## Results

### Clinical features and outcomes

Mice injected with LPS displayed clinical features including decreased activity, somnolence, hunched appearance, piloerection, ocular discharge, hypothermia, and weight loss (Table 2).

**Table 2.** Change in weight (g) of mice 6, 24 and 48 h after an injection of saline or LPS.

	Saline	LPS
6 h	−0.35 (±0.08)	−0.7 (±0.05)**
24 h	+ 0.42 (±0.36)	−1.91 (±0.12)***
48 h	+ 0.37 (±0.27)	−2.01 (±0.44)***

Mean ± SEM.

** *p* ≤ 0.01.

*** *p* ≤ 0.001.

Body temperature dropped significantly at 6 h (33.1 ± 0.4℃; *t* = 6.611, df = 40.710 (adjusted for inequality of variance), *p* < 0.001) and 24 h (33.0 ± 0.7℃; *t* = 4.381, df = 19.757 (adjusted for inequality of variance), *p* < 0.001), but not at 48 hours (35.6 ± 0.6℃; *t* = 0.722, df = 23, *p* = 0.477) after LPS injection as compared with saline controls (35.9 ± 0.1℃). A positive correlation was seen between weight loss and temperature at 24 h after LPS injection (*r* = 0.798, df = 17, *p* < 0.001; Spearman’s rank correlation coefficient) with more weight loss evident in warmer mice.

**Figure 1.**
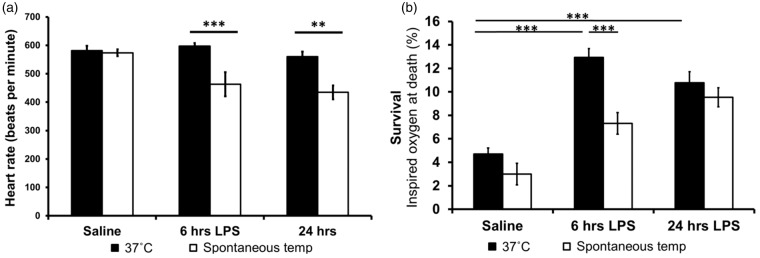
(a) No change in heart rate was seen at 6 or 24 h post-injection of LPS in normothermic mice. However, at their spontaneous temperature, endotoxic mice had a significantly lower heart rate. **p ≤ 0.01, ***p ≤ 0.001. (b) Mortality of mice subjected to systemic inflammation/saline and hypoxemia. Data are displayed as mean ± SEM.

When temperature was controlled at 37℃, heart rate did not differ between LPS- and saline-injected mice. However, spontaneous hypothermia reduced heart rate significantly at both 6 h (*t* = 4.548, df = 9.171 (adjusted for inequality of variance), *p* = 0.001) and 24 h (*t* = 2.950, df = 18, *p* = 0.009) after LPS injection (Figure 1a).

**Figure 2.**
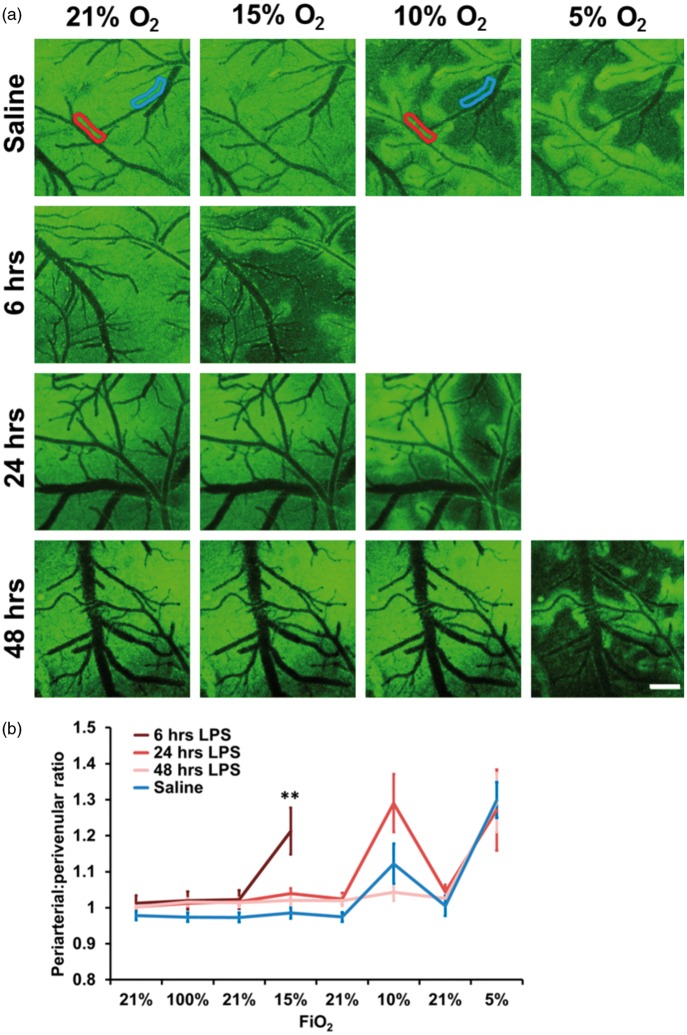
The sensitivity of flavoprotein signal to changes in inspired oxygen is affected by systemic inflammation. (a) Flavoprotein signal changes in endotoxic and control mice with changes in inspired oxygen. Blue = representative selection of perivenular tissue; red = representative selection of periarterial tissue. Scale bar is 200 µm. (b) Quantification of the change in endogenous flavoprotein fluorescence around veins relative to arteries. Data are displayed as mean + SEM. **p (saline vs 6 h LPS) ≤ 0.01.

Survival during decreased FiO_2_ was reduced in LPS-injected mice compared with saline-injected controls, but spontaneous hypothermia significantly improved resilience to increased levels of hypoxemia at 6 h post-LPS injection (Figure 1b): during progressive hypoxemia, LPS-injected mice died at higher FiO_2_ values compared to saline-injected controls [*t* = 9.050, df = 44.291 (adjusted for inequality of variance), *p* < 0.001]. However, hypothermia (both *spontaneous* and *induced*) improved tolerance to hypoxemia in endotoxic mice 6 h after LPS (*t* = 4.803, df = 28, *p* < 0.001 and *t* = 2.868, df = 28, *p* = 0.008, respectively) (Figure 1b).

### Flavoprotein redox state

We previously reported that healthy animals subjected to hypoxemia showed a characteristic change in the distribution of cortical flavoprotein fluorescence, exposing areas of vulnerability, revealed by decreased fluorescence (which reflects a more reduced state of the flavoprotein pool) distal to arteries and adjacent to veins. However, flavoprotein fluorescence intensity was preserved near arteries (Figure 2), forming an *‘arterial halo’ pattern*. In animals injected with normal saline, this arterial halo pattern appeared at the same FiO_2_, regardless of the time from injection (data not shown) and therefore data from mice injected with saline were pooled. On breathing room air, the flavoprotein signal covered the cortex uniformly with no difference between periarterial and perivenular fluorescence, and this did not change when FiO_2_ was increased to 100% in all groups (Figure 2b). In saline-injected animals, exposure to 15% inspired oxygen also failed to alter the distribution of the cortical flavoprotein signal. However, on decreasing the inspired oxygen to 10%, a change in cortical flavoprotein fluorescence was seen in 11 of 21 saline-injected mice with the appearance of the characteristic arterial halo pattern*.* All but two of the remaining animals showed this pattern when FiO_2_ was further reduced to 5% (Figure 2).

In contrast, at 6 h post-injection, the arterial halo pattern appeared in the majority (9/17) of animals injected with LPS even when breathing 15% O_2_ (Figure 2). At 24 h post-LPS, this sensitivity of the flavoprotein signal to changes in inspired oxygen was attenuated: the arterial halo pattern appeared at FiO_2_ values between 0.10 and 0.15 in 77% of animals (10/13; Figure 2). By 48 h post-LPS, clinical signs, survival and flavoprotein sensitivity to changes in inspired oxygen did not differ from saline-injected controls (Figures 1(b) and 2).

Changes in flavoprotein fluorescence with hypoxemia were reversible when FiO_2_ was returned to 0.21 in all surviving animals. Here, the periarterial flavoprotein signal expanded until it again covered the cortex uniformly and the ratio of periarterial to perivenular fluorescence returned back to ∼1 (Figure 2b).

### Cortical tissue oxygenation

**Figure 3.**
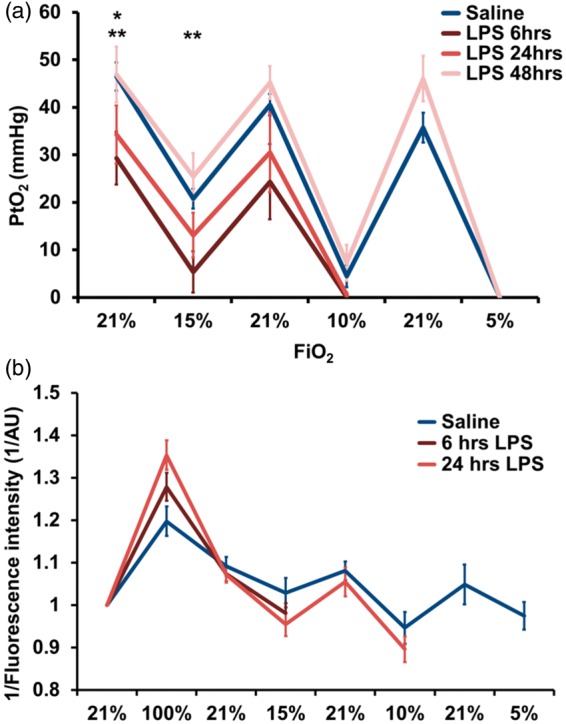
Oxygenation of the cortex during changes in inspired oxygen (FiO_2_). (a) Oxygen sensor measurements of cortical oxygenation. (b) Relative changes in oxygen (shown as reciprocal of fluorescence intensity of the sensor bead) with changes in FiO_2_. Data displayed as mean ± SEM. **p (saline vs 6 h LPS) ≤ 0.01, *p (saline vs 24 h LPS) ≤ 0.05; one sided *t*-test.

The cortical PtO_2_ of saline-injected control mice while breathing room air was higher (46.5 ± 3.0 mmHg) than mice injected with LPS at both 6 h (29.3 ± 5.6 mmHg; *t* = 3.044, df = 7, *p* = 0.01, single tailed *t*-test) and 24 h (34.3 ± 6.1 mmHg; *t* = 2.071, df = 7, *p* = 0.039, single tailed *t*-test). This pattern persisted at lower FiO_2_ although the differences were not always significant (Figure 3a). At 48 h, the cortices of LPS-injected animals had recovered to control PtO_2_ levels (46.9 ± 5.9 mmHg, Figure 3a). A reduced cortical PtO_2_ on decreasing the FiO_2_ was confirmed in imaging experiments using oxygen-sensitive phosphorescent microbeads (Figure 3b).

### Cortical redox changes in response to hypoxemia during hypothermia (spontaneous or induced)

**Figure 4.**
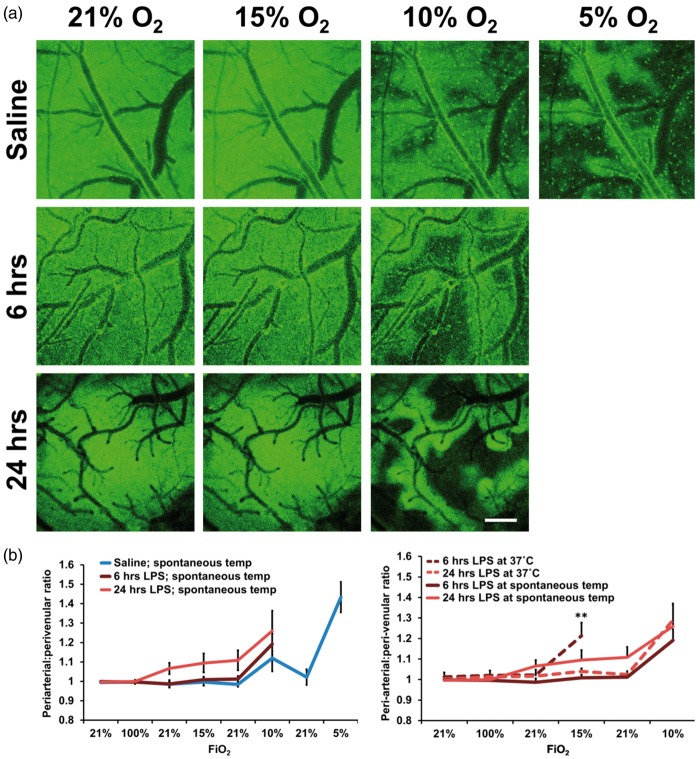
Spontaneous hypothermia protects cortical mitochondria from hypoxemia. (a) Changes in cortical flavoprotein signal during changes in FiO_2_ in saline- and LPS-injected animals kept at their spontaneous temperature. (b) Quantification of the ratio of periarterial over perivenular fluorescence in saline- and LPS-injected animals kept at their spontaneous temperature and at 37℃. Data are displayed as mean ± SEM. **p (6 h LPS 37℃ vs spontaneous temperature) ≤ 0.01.

The rectal temperature of saline-injected mice was 35.9 ± 0.1℃. The arterial halo pattern response to differences in FiO_2_ in these mice was very similar to saline-injected mice kept at 37℃ (see above). The mean temperature in LPS-injected mice was significantly lower at 6 h after injection [33.1 ± 0.4℃; *t* = 6.611, df = 40.710 (adjusted for inequality of variance), *p* < 0.001]. If this spontaneous hypothermia was maintained during imaging, the cortical mitochondria were protected relative to normothermic (37℃) LPS-injected mice, such that the observed arterial halo pattern response to changes in FiO_2_ was similar to saline-injected controls (Figure 4), accompanied by an improvement in survival during hypoxemia (Figure 1b). However, this protective effect was not seen at 24 h after injection, despite the LPS-injected mice still having a lower baseline mean temperature (33.0 ± 0.7℃) than saline-injected controls (Figures 1(b) and 4). However, it should be noted that fewer normothermic (37℃), LPS-injected animals survived surgery 24 hours after injection compared to those in which spontaneous hypothermia was maintained. Data from this group of animals were not included as no imaging data could be collected.

**Figure 5.**
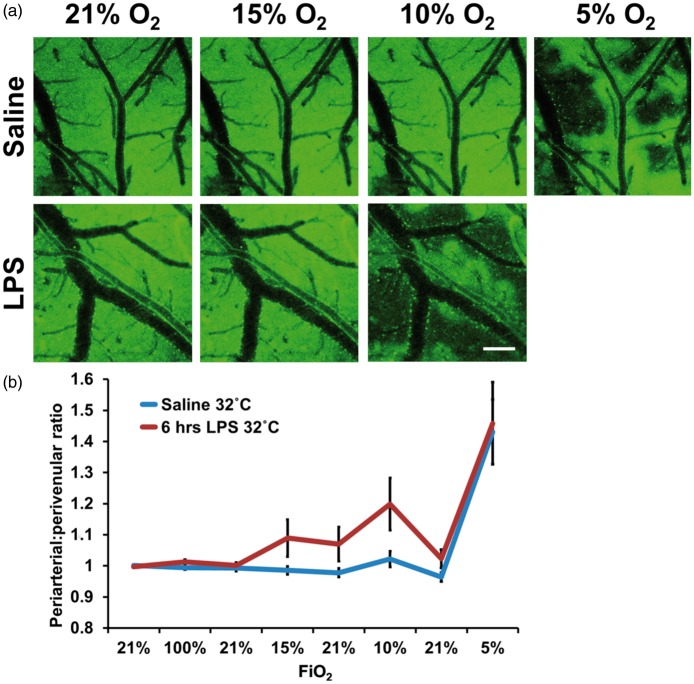
Forced hypothermia protects cortical mitochondria from hypoxemia. (a) Changes in cortical flavoprotein signal during changes in FiO_2_ in saline- and LPS-injected animals 6 h after endotoxemia kept at 32℃. (b) Quantification of the ratio of periarterial over perivenular fluorescence. Data are displayed as mean ± SEM.

As we observed a protective effect of hypothermia in LPS-injected animals at 6 h, we sought to determine the effects of hypothermia on saline-injected controls. Induced hypothermia (32℃) was protective for both saline- and LPS-injected mice (Figure 5) with mortality and the arterial halo pattern response not appearing until a lower FiO_2_ was administered (Figure 5): only 53% of normothermic animals (9/17) survived on reducing inspired oxygen to 15% at 6 h post-LPS compared to all but one surviving (12/13) when kept under induced hypothermia, and 100% (13/13) surviving when spontaneous hypothermia was maintained.

## Discussion

We report that in mice, endotoxemia temporarily increases mortality and cortical mitochondrial vulnerability (as assessed by flavoprotein fluorescence) in response to hypoxemia, and that these LPS-induced vulnerabilities are enhanced when animals are forcibly kept at an increased core body temperature of 37℃.

Bioenergetic failure is postulated to be an important cause of multiple organ dysfunction in sepsis.^36^ Here we investigated whether an additional bioenergetic challenge, namely hypoxemia, promotes further mitochondrial dysfunction in the cortices of mice subjected to mild systemic inflammation. We report, in agreement with previous work, that mitochondrial dysfunction, defined by a loss of flavoprotein signal (i.e. increased reduced state of the flavoprotein pool^37^), is very pronounced during hypoxemia, revealing areas of vulnerability in the cortex between vessels and surrounding veins, yet with protected ‘*halos*’ close to arteries in which the flavoprotein pool remains oxidised.^33,38^ Importantly, areas of vulnerability and increased mortality were observed at higher concentrations of inspired oxygen in animals during systemic inflammation. This effect was most pronounced at 6 h after LPS injection but had returned to control levels by 48 h. The present findings suggest that either mitochondrial dysfunction and/or organ hypoperfusion (macro and/or microvascular) is involved in the pathology of endotoxemia.

Previous reports of microvascular disturbances in sepsis^15–17,23,24^ and our own observation that mitochondrial vulnerability in the endotoxic brain conforms precisely to the vascular anatomy, strongly implicate compromised oxygen availability and/or altered mitochondrial oxygen consumption. Therefore, we measured oxygen within the cortex, revealing that cortical tissue oxygen tension was lower in endotoxic animals than healthy controls. The lowest values of cortical tissue oxygen were measured 6 h after LPS injection with a partial recovery by 24 h, and returned to control levels by 48 h after LPS injection. This coincided with the increased vulnerability of LPS-injected mice to hypoxemia, and supports evidence implicating microvascular dysfunction as a leading cause of multiple organ dysfunction.^15–17,23,24^ As a limitation of this model, fluid resuscitation was not given so underlying hypovolemia in the endotoxic mice may have exacerbated the impact of the hypoxemic insult. Changes in cerebral oxygenation in response to reductions in FiO_2_ were confirmed in imaging experiments using oxygen-sensitive microbeads.

The literature on the effect of therapeutic hypothermia in sepsis is conflicting. Thus, hypothermia has shown neuroprotective potential in several intensive care conditions (for review see Bernard and Buist^29^), while a recent study of controlled hypothermia in patients with meningitis was stopped due to increased mortality.^31^ In mice, the septic phenotype, unlike that seen in patients, is one of hypothermia with an associated increase in illness severity, metabolic shutdown and mortality.^32^ We hypothesized that reducing metabolic demand through hypothermia would protect both endotoxic and healthy mice during the additional bioenergetic challenge of hypoxemia. Indeed, this was the case for both spontaneous and controlled (32℃) hypothermia where improved preservation of mitochondrial flavoprotein fluorescence and survival was seen at 6 h after LPS injection. Instead, the forced recovery of endotoxic mice to normothermia was detrimental during progressive hypoxemic insults, suggesting that the temperature-induced increase in whole body metabolism could not be adequately matched by an increase in mitochondrial ATP production. Many species of mice hibernate in prolonged cold conditions, so the hypometabolic response to a severe inflammatory stress (in this case endotoxemia) may represent, at least in milder forms of sepsis, a specific adaptive phenomenon in this species.^39,40^

Extrapolating the present findings to a human population should be done with caution as endotoxemia initiated by a single LPS injection causes an inflammatory condition which is not pathogen-driven, and the animals in these studies received no resuscitation. Therefore, further studies are needed to determine whether the present findings are replicated in other models of sepsis.

In conclusion, we report that systemic inflammation leads to increased sensitivity of cortical mitochondria to hypoxemia and that this increased sensitivity is mirrored by a decrease in cortical tissue oxygen tension. Having revealed a bioenergetics supply–demand imbalance in this endotoxic model, we also show that reducing metabolic demand through hypothermia increased survival and reduced the vulnerability of cortical mitochondria during severe hypoxemia.
